# A Fully Implantable Miniaturized Liquid Crystal Polymer (LCP)-Based Spinal Cord Stimulator for Pain Control

**DOI:** 10.3390/s22020501

**Published:** 2022-01-10

**Authors:** Seunghyeon Yun, Chin Su Koh, Jungmin Seo, Shinyong Shim, Minkyung Park, Hyun Ho Jung, Kyungsik Eom, Jin Woo Chang, Sung June Kim

**Affiliations:** 1 Department of Electrical and Computer Engineering, College of Engineering, Seoul National University, Seoul 08826, Korea; shy5812@gmail.com (S.Y.); jmseo.16@gmail.com (J.S.); simsinyong@gmail.com (S.S.); kimsj@snu.ac.kr (S.J.K.); 2 Department of Neurosurgery, College of Medicine, Yonsei University, Seoul 03722, Korea; CSKOH@yuhs.ac (C.S.K.); mkpark3142@yuhs.ac (M.P.); JUNGHH@yuhs.ac (H.H.J.); 3 Department of Electronics Engineering, College of Engineering, Pusan National University, Busan 46241, Korea

**Keywords:** spinal cord stimulation, liquid crystal polymer, implantable device, pain control

## Abstract

Spinal cord stimulation is a therapy to treat the severe neuropathic pain by suppressing the pain signal via electrical stimulation of the spinal cord. The conventional metal packaged and battery-operated implantable pulse generator (IPG) produces electrical pulses to stimulate the spinal cord. Despite its stable operation after implantation, the implantation site is limited due to its bulky size and heavy weight. Wireless communications including wireless power charging is also restricted, which is mainly attributed to the electromagnetic shielding of the metal package. To overcome these limitations, here, we developed a fully implantable miniaturized spinal cord stimulator based on a biocompatible liquid crystal polymer (LCP). The fabrication of electrode arrays in the LCP substrate and monolithically encapsulating the circuitries using LCP packaging reduces the weight (0.4 g) and the size (the width, length, and thickness are 25.3, 9.3, and 1.9 mm, respectively). An inductive link was utilized to wirelessly transfer the power and the data to implanted circuitries to generate the stimulus pulse. Prior to implantation of the device, operation of the pulse generator was evaluated, and characteristics of stimulation electrode such as an electrochemical impedance spectroscopy (EIS) were measured. The LCP-based spinal cord stimulator was implanted into the spared nerve injury rat model. The degree of pain suppression upon spinal cord stimulation was assessed via the Von Frey test where the mechanical stimulation threshold was evaluated by monitoring the paw withdrawal responses. With no spinal cord stimulation, the mechanical stimulation threshold was observed as 1.47 ± 0.623 g, whereas the stimulation threshold was increased to 12.7 ± 4.00 g after spinal cord stimulation, confirming the efficacy of pain suppression via electrical stimulation of the spinal cord. This LCP-based spinal cord stimulator opens new avenues for the development of a miniaturized but still effective spinal cord stimulator.

## 1. Introduction

Spinal cord stimulation is a therapeutic tool used to treat certain chronic pain conditions especially related to neuropathic pain caused by trauma or postoperative sequelae and sensory nerve damage [1,2,3]. The idea was to deliver electric current pulses to the spinal cord through an electrode inserted into the fat layer of the spine [4]. Based on the well-known gate control theory, the stimulation current delivered to the spinal cord inhibits the transmission of the nociceptive information as well as other sensory information [5,6,7,8]. Since the effects of pain suppression persist only while the stimulation is being delivered, an implantable spinal cord stimulator that allows continuous activation of the spinal cord to alleviate the pain is crucial for the patients suffering chronic neuropathic pain.

The efficacy of spinal cord stimulation has been demonstrated in several clinical studies, and commercial spinal cord stimulators have been developed for human use [9,10,11,12]. Medtronic developed a spinal cord stimulator that includes stimulation electrodes and a universal implantable pulse generator (IPG; PrimeAdvanced, Medtronic, Minneapolis, MN, USA). The titanium packaged IPG operated by a battery produces stimulation pulses via a constant voltage scheme whose stimulation amplitude and frequency lies in the range from 2 to 130 Hz and from 0 to 10.5 V, respectively [13]. Stimulation pulses are delivered through the polyurethane-insulated Pt-Ir electrodes of a wire diameter of 1.3 mm [14]. Nevro Corporation have developed a spinal cord stimulation system, Senza System, which consist of two eight-channel percutaneous electrodes, an IPG, and an anchor. Upon current stimulation, neurons in the target area are desensitized resulting in the local paresthesia, a tingling sensation, and finally masking the pain without interlocking with other senses [9]. Although the stimulation rate used in this system lies in the kilohertz region, far from the widely employed stimulation rate of 40 to 60 Hz [15,16,17], the pain reduction efficacy has been proven in clinical trials. Al-Kaisy et al. reported that 88% (72 out of 82) of patients showed a significant improvement in pain score [18], and Kapural et al. demonstrated that kilohertz spinal cord stimulation effectively reduced the pain even without paresthesia [10].

Despite their successes in alleviating the chronic pain, these devices have several limitations, which originated from the following features. Since IPGs are packaged by the metal, its volume and the weight are fairly bulky and heavy (i.e., the volume and the weight of the PrimeAdvanced are around 40 cm^3^ and 70 g, respectively), leading to various complications after implantation as well as restriction in the implantation location (i.e., hip or abdomen) [4,13,19]. Since the location of the implanted IPG is distant away from the actual stimulation position, a long lead wire is required to connect the electrode and the IPG [13,17], which results in complicated surgery for making a tunnel under the skin and risk of infection of a widespread lead wire. The use of metal as a packaging material raised a problem of electromagnetic shielding impeding the electromagnetic (EM) waves for wireless power and data transmission [20,21]. Moreover, an excessive heat is generated at the metal package when it exposed to the strong alternating magnetic field especially during the magnetic resonance (MR) imaging [22,23,24]. Since a lead wire is encapsulated by a silicone whose moisture absorption rate (>1%) is significantly higher than that of the metal, the moisture can infiltrate through the gap between the electrode and the circuit, and in the worst case, the circuit as well as the human may be at risk.

LCP has been widely employed as an insulating and substrate material for not only in the implantable devices but also in the microwave integrated circuit due to its biocompatibility and low moisture absorption rate (<0.04%) compared to other polymers such as polyimide, parylene, or silicon elastomer [25,26,27,28]. Moreover, due to its flexible but strong mechanical properties, the LCP-based neural probe does not require a guiding post to pierce the dura, a rigid sheath covering the brain, during brain insertion [29,30]. Previous studies have demonstrated the fabrication process of LCP-based neural probes via semiconductor processes where the LCP is employed as both the substrate and encapsulation material [29,31]. With the exception of small electrodes and metal wires, LCP-based neural probes are composed of the LCP polymer allowing EM waves to penetrate without seriously damping its signal for wireless power and data delivery [32,33].

In this study, we developed a fully implantable neural stimulator using LCP for spinal cord stimulation. Unlike conventional spinal cord stimulators employing metal-based packaging and manually assembled electrode array, the developed spinal cord stimulator uses the lightweight and semi-hermetic LCP to develop micro-fabricable and ultrathin electrode arrays and to monolithically encapsulate the device. An inductive link was utilized to transmit power and data to the implanted circuitry. A pulse generation circuit and microfabricated electrode arrays are monolithically integrated and packaged. The fabricated stimulator was evaluated in vivo to demonstrate the pain suppression effect of the spinal cord stimulator.

## 2. Materials and Methods

### 2.1. System Overview

**Figure 1:**
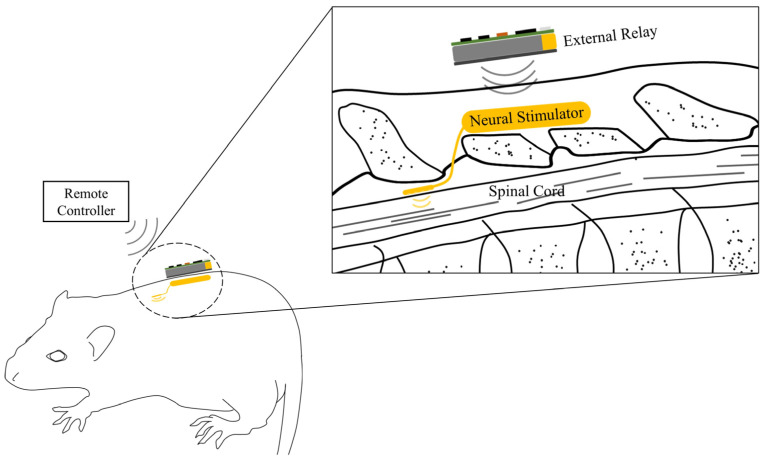
A fully implantable neural stimulation system. The neural stimulator consisting of an implantable pulse generator and stimulation electrodes are located outside of the vertebra and at the epidural part of the spinal cord, respectively. The external relay is attached on the skin to wirelessly transfer the power and data to the implanted neural stimulator.

The spinal cord stimulator system consists of an implanted neural stimulator and two external devices: a skin attached external relay and a remote controller (Figure 1). Stimulation protocols are preset at the controller and delivered to the external relay via Zigbee communication [34]. Then, the external relay transfers received stimulation protocols and power to the implanted neural stimulator via an inductive link. The neural stimulator consisting of an electrode array, a receiver coil, and an application-specific integrated circuit (ASIC) circuitries generating biphasic current pulses were implemented and monolithically packaged using a flexible LCP layer. The neural stimulator located outside of the vertebra generates electrical stimulation pulses and send them to the microfabricated electrodes implanted at the epidural part of the spinal cord to stimulate target neurons (Figure 1).

### 2.2. Neural Stimulator

#### 2.2.1. Electrode Array

Electrode arrays were microfabricated on LCP films [30,35]. Briefly, the LCP film was first attached onto the 4-inch silicon wafer using an elastomer. The elastomer (MED 6233, Nusil Silicone Technology, Carpinteria, CA, USA) acting as a glue was spin-coated on the wafer. A 25 μm-thick LCP film with high melting temperature (HT-LCP; 330 °C, Vecstar CT-Z series, Kuraray, Japan) was laser cut into 4-inch-wafer-sized pieces using a UV laser system (Samurai System, DPSS, Los Angeles, CA, USA). The cut LCP piece was attached to the elastomer coated silicon wafer using a roller. As for the first procedure of metal patterning, the LCP substrate was placed under O_2_ plasma for 180 s using an etcher (Oxford Etcher 80Plus, Oxford Instruments, Abingdon, UK). Subsequently, a 50 nm-thick titanium layer and a 100 nm-thick gold layer were sequentially deposited on the LCP substrate by evaporation. A photoresist layer (AZ4620, Clariant, Muttenz, Switzerland) was spin-coated on the gold layer and patterned using an aligner (MA6/BA6, SUSS MicroTec, Garching, Germany) and a developer (AZ300, AZ Electronic Materials, Luxembourg). An 8 μm-thick gold layer was electroplated on the areas without photoresist followed by removing the photoresist by applying it using a striper (AZ700, AZ Electronic Materials, Luxembourg). The remaining thin metal layers on the LCP were etched with aqua regia and HF. Finally, the outlining process was performed by lasers machining using the UV laser system for post-lamination processes.

#### 2.2.2. ASIC Circuitries and Receiver Coil

The ASIC circuitries for the implanted neural stimulator is capable of wireless power and data transmission, voltage level adjustment, and neural stimulation. The data containing desired stimulation parameters are fed into the internal device via an inductive link operated at 2.5 MHz. To convert the pulse-width modulated (PWM) data to half-sinusoidal signals, the wirelessly received data are fed into a resistor and a 3.3 V Zener diode. Based on half-sinusoidal signals, an ASIC generates biphasic electrical stimulation pulses whose stimulation frequency, duration, and current magnitude are in the range of 20 to 230 Hz, 10 to 630 μs, and 0.01 to 10.23 mA, respectively. Along with the data transmission, 3.3 V constant voltage is produced to power an oscillator (LTC6906, Linear Technology, Milpitas, CA, USA) and the digital circuitry of the ASIC. Pulse-width modulated signals are first rectified at the rectifier which are then fed into the Zener diode connected with a capacitor to clamp the voltage to 15 V. Finally, 15 V is used to create 3.3 V constant voltage, which is produced at the regulator (NCP71533, On Semiconductor, Phoenix, AZ, USA). The ASIC circuitries were fabricated on the 100 μm-thick copper-cladded LCP films (HT-LCP, ULTRALAM 3850, Rogers Corporation, Chandler, AZ, USA) whose melting temperature was the same as that of the LCP film used in the electrode array layer. A stimulation generator ASIC was wire-bonded, and the other auxiliary electronics including a regulator, Zener diode, oscillator, and rectifier were mounted using conductive epoxy and baked in the oven at 100 °C for 1 h to harden the epoxy.

We designed a spirally wound and rectangular-shaped receiver coil whose outer width, outer length, inner width, inner length, line width, number of turns, and number of layers are 18 mm, 6 mm, 13 mm, 1 mm, 100 μm, 26 turns, and 2 layers, respectively. Analogous to the ASIC circuitries, the receiver coil is fabricated on the 100 μm-thick copper-cladded HT-LCP films (ULTRALAM 3850, Rogers Corporation, USA). The tuning capacitor was connected in parallel with respect to the receiver coil to form a parallel resonance circuit to maximize the power transmission efficiency.

#### 2.2.3. Monolithic Integration and Packaging

**Figure 2:**
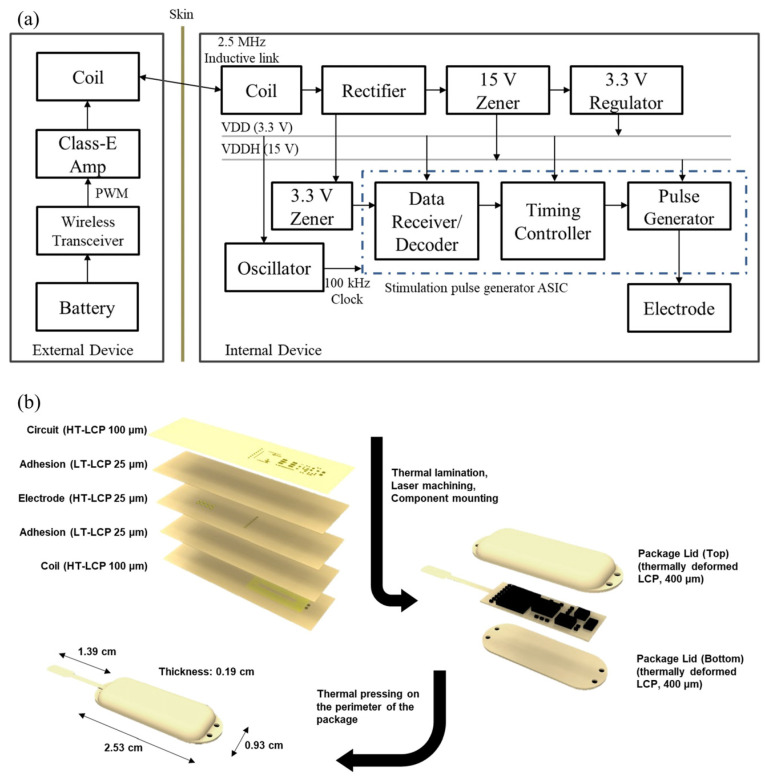
(**a**) A block diagram of the neural stimulator and the external relay. The internal device receives power and data from the external relay through 2.5 MHz inductive link to generate a biphasic pulse. (**b**) Fabrication process of the designed neural stimulator. Microfabricated electrode/circuit layers are thermally laminated, laser machined, and electrical components are mounted before being packaged with customized LCP (liquid crystal polymer) lids. HT-LCP: high melting temperature LCP, LT-LCP: low melting temperature LCP.

The layers of ASIC circuitries, electrode arrays, and coil were sequentially stacked. As adhesive layers, 25 μm-thick LCP films with a low melting temperature (LT-LCP; 280 °C, Vecstar CT-F series, Kuraray, Tokyo, Japan) were placed in between each adjacent layer (Figure 2). All the films were aligned in a pair of custom-designed aluminum jig and laminated at 285 °C for 10 min with a load of 2 kg/cm^2^ in a heat press (Model 4330, Carver, Wabash, IN, USA). Two via holes were made at the markers on the ASIC circuitries and electrode array layers by the laser machining. The via holes were filled with conductive epoxy (H20E, Epotek, Billerica, MA, USA) to connect coil to the ASIC circuitries. Prior to the packaging process, a series of laser-machining (Samurai System, DPSS, USA) processes including electrode site opening and an outlining process were performed.

Two lids were fabricated to encapsulate the system. Briefly, 100 μm thick LT- and HT-LCP films were laser cut to have dimensions of 20 mm × 40 mm. Two HT-LCP films and two LT-LCP films were stacked one after another to form a pair of LCP layers. Two pairs of LCP layers were aligned using custom-designed aluminum jigs of which to make top and bottom package lids. One pair of jigs was designed to have curvature to shape the top package lid. These LCP layers were thermally laminated together and deformed using a heat press (290 °C for 10 min with a load of 2 kg/cm^2^) to form 400 μm thick top and bottom package lids in desired shapes, as shown in Figure 2b.

The assembled stimulator circuit board was placed in between the two package lids with the electrode extruded at the center of a rounded lid corner. The circuit board was fixed at the bottom lid by locally applied heat at the rim of the board. This was followed by thermal lamination on the edge of the package with a 2 mm margin for biocompatible encapsulation of the electronics to protect them from body fluids. The edge of the package was trimmed by a laser.

### 2.3. External Devices

The stimulation parameters (the channel configuration, the pulse amplitude, the pulse width, and the pulse rate) and ON/OFF commands are adjusted at the remote controller and wirelessly delivered to the external relay via two Zigbee-compliant RF transceivers (CC2530, Texas Instrument, Dallas, TX, USA) implemented on either side of external devices [34]. Once receiving the data from the remote controller, the wireless transceiver at the external relay tailors the stimulation parameters and controls the class-E amplifiers by generating pulse-width modulated (PWM) data from the general-purpose input/output (GPIO). The class-E amplifier along with the transmitter coil was driven by a PWM-modulated 2.5 MHz sinusoidal wave to deliver the power and data to the internal stimulation ASIC via inductive link. A 1300 mAh lithium battery (TW-103440, The Han, Korea) is used to supply the power to the external relay.

### 2.4. System Evaluation

The performance of the spinal cord stimulator system was evaluated in bench. First, the overall operation of the system was monitored to confirm whether desired stimulation pulses were generated. After setting the stimulation parameter at the remote controller, the output voltage of the regulator was monitored to validate the power delivery, and the ASIC chip generated stimulation waveform was monitored to evaluate the overall system operation. During the evaluation, the distance between the remote controller to the external relay was kept within the reliable transmission range, and the external relay was separated by 5 mm apart from the neural stimulator. Next, the performance of the stimulation electrode was evaluated to confirm whether the generated stimulation pulse can be delivered to the neural tissue. The electrochemical impedance spectroscopy (EIS) and cyclic voltammetry (CV) of the fabricated electrode arrays were measured with a potentiostat (SI 1287/SI 1260, Solatron, Hampshire, UK) configured as a three-electrode cell using an Ag/AgCl reference electrode and a Pt counter electrode in PBS solution (1X, Gibco). The EIS was measured with a frequency ranging from 1 Hz to 100 kHz by applying 10 mV of sinusoidal wave. The CV curve was obtained by monitoring the current flowing through the electrode array while sweeping the voltage in the range of −0.8 to 0.6 V.

### 2.5. Animals and In Vivo Evaluation Setup

A total of 5 adult male Sprague–Dawley rats (Orientbio Inc., Osan, Korea) weighing in the range from 200 to 250 g were used in this study. Rats were individually housed in polycarbonate cages with wood chip bedding while free access to food and water was allowed. A 12 h light/dark cycle (light: 08:00–20:00) was maintained with a housing temperature and humidity of 24 ± 2° and 55 ± 5%, respectively. The Institutional Animal Care and Use Committee of Yonsei University (IACUC no. 2016-0161, date of approval 26 September 2016) approved the study. A spared nerve injury (SNI) model was chosen as a neuropathic pain model [36]. Rats were anesthetized via an intraperitoneal injection of pentobarbital (40 mg/kg) (Hanlim Pharm, Yongin, Korea). The rats were injected with 0.1 mL of atropine (Huons, Seongnam, Korea) 10 min before the pentobarbital injection to ensure the stability of anesthesia. The left sciatic nerve was exposed, and three major divisions of the sciatic nerve (tibial, sural, and common peroneal nerve) were clearly separated. To generate an efficient neuropathic pain model, the common peroneal and tibial nerves were completely ligated and transected, leaving the sural nerve intact. Hemostasis was completed, and the cut was closed with muscle and skin sutures. One week after surgery, we measured the pain threshold to confirm whether neuropathic pain has been induced. After a behavioral test, rats that did not show neuropathic pain response were excluded from this study. To implant the spinal cord neural stimulator (SCNS), rats were anesthetized with pentobarbital sodium and a midline skin incision was made, and laminectomy was performed to access the spinal cord. The SCNS electrode was implanted epidurally between thoracic 13 (T13) and lumbar 1 (L1) level. Caution was taken to avoid penetrating the dura while inserting the electrode. The package part connected to the electrode was placed in line with the vertebra and fixed with sutures. The wounds were closed with sutures, and the stimulation test commenced three days thereafter.

Rats were placed inside acrylic cages (8 cm × 10 cm × 20 cm) on a wire mesh grid, and the external relay was attached to the back of the rats. Innocuous mechanical stimulation was applied with a von Frey filament to the lateral edge of the left hind paw to measure the thresholds of the flexion withdrawal reflex in response to mechanical stimulation of the hind limb. As a control experiment, the threshold was measured while no electrical stimulation was applied. To modulate the pain signal by electrical stimulation, the biphasic current stimulation pulses were delivered to the spinal cord having the pulse rate and the duration of 38.8 Hz and 120 μs, respectively. To confirm the efficacy of electrical spinal cord stimulation for pain-relieving effects, the mechanical withdrawal threshold was measured followed by four different stimulation parameters (pre-stimulation, 100, 250, and 500 μA). Electrical stimulation was maintained for 15 min for each parameter, and a von Frey test was performed afterwards. To remove the effect of the previous test, the next electrical stimulation was delivered at an interval of one hour. Rats were sacrificed after all experiments, and the spinal cord stimulation device was removed from the rats.

### 2.6. Statistical Analysis

All data are presented as the mean ± standard error of the mean (SEM). GraphPad Prism 5 (GraphPad Software, Inc., La Jolla, CA, USA) was used to create graphs and perform all statistical analyses. Repeated-measures ANOVA followed Dunnett’s post hoc were used to analyze the mechanical threshold changes induced by spinal cord stimulation.

## 3. Results

### 3.1. Fabricated System

**Figure 3:**
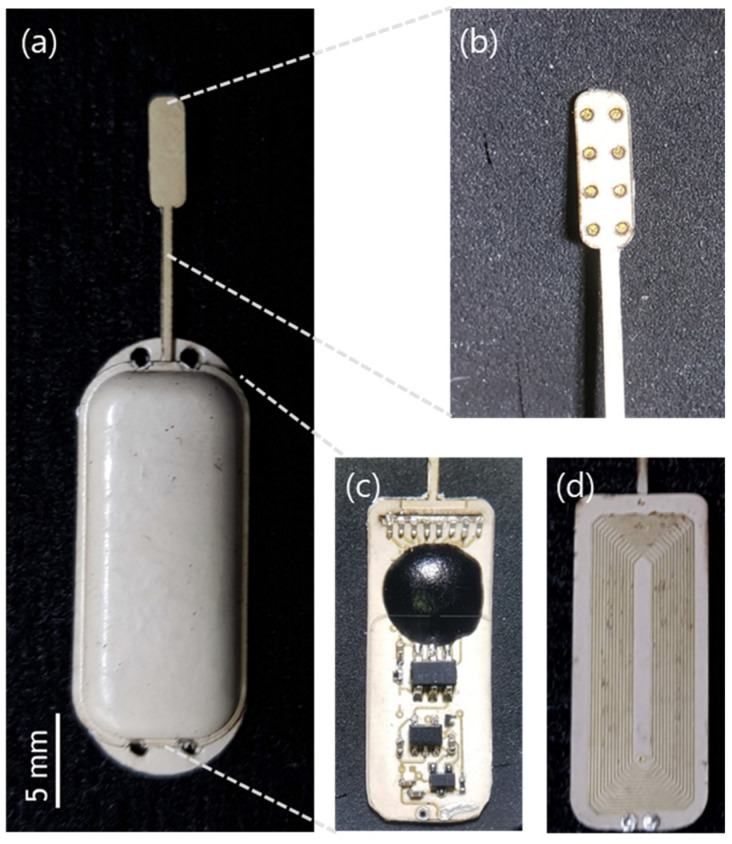
(**a**) Fabricated implantable neural stimulator; (**b**) Electrode part; (**c**) Circuit layer; and (**d**) coil layer in the package.

The final dimension of the fabricated neural stimulator without extruded electrode in the width, the length, and the thickness are 2.53 cm, 0.93 cm, and 0.19 cm, respectively (Figure 3). The total weight of the neural stimulation is only 0.4 g. Considering the dimension and the weight of the rat, this miniaturized neural stimulator would allow the freedom of implantation location and reduce the discomfort of animal after implantation. To stimulate the spinal cord, the electrode sites were opened on the bottom side of the neural stimulator with a site area of 0.442 mm^2^ each. The shank was elongated having the final length of electrode of 13.9 mm with a total electrode length and thickness of 700 μm and 100 μm, respectively.

### 3.2. System Evaluation

**Figure 4:**
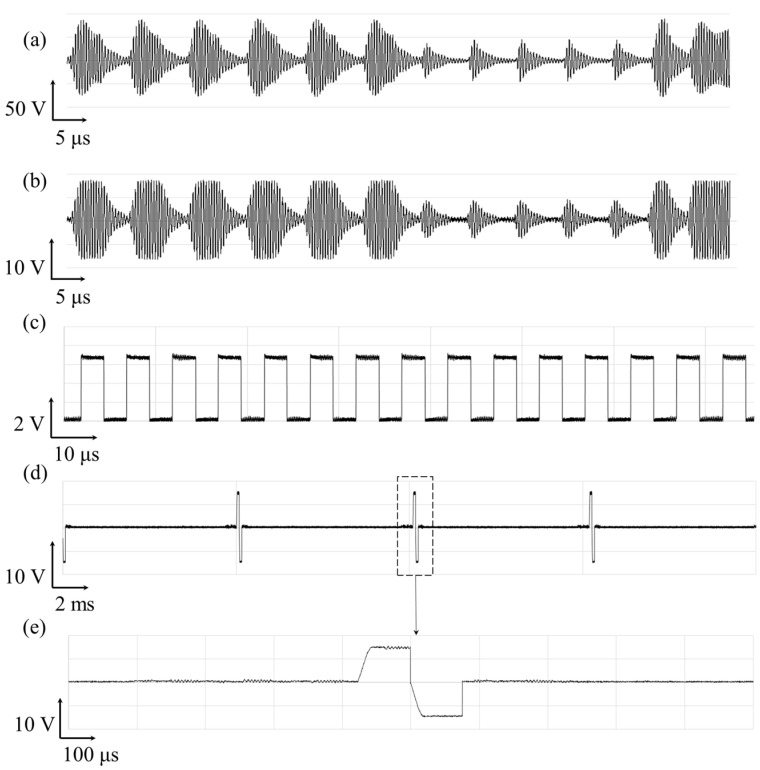
Exemplar waveform of the wireless operation of spinal cord stimulator and its stimulation current pulse generation. The voltage measured across the (**a**) the transmitter coil and (**b**) the receiver coil; the output voltage at the (**c**) oscillator; and (**d**,**e**) stimulation generator ASIC.

The exemplar waveforms measured at the assembled external relay and IPG are shown in Figure 4. The PWM signal modulated by a 2.5 MHz carrier wave is fed into the transmitter coil (Figure 4a) and wirelessly delivered to the receiver coil (Figure 4b). A 240-μF tuning capacitor was connected in parallel to the receiver coil to maximize the voltage across the receiver coil. The output voltage at the oscillator fed into the ASIC was measured (Figure 4c). Finally, stimulation waveforms were measured at the ASIC (Figure 4d,e). We confirmed that the sufficient power and valid data were delivered to the ASIC chip to produce a series of biphasic stimulation current pulses.

**Figure 5:**
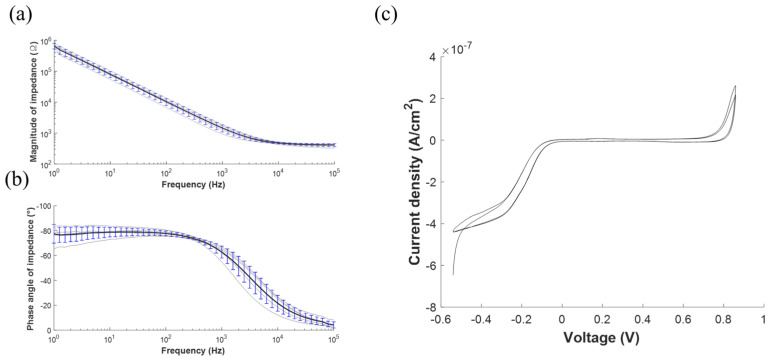
Electrochemical characterization of the stimulation electrodes array. (**a**) Electrochemical impedance spectroscopy (EIS) measurements as represented by the mean (black line) and the standard deviation (blue bar) of the (**a**) magnitude and the (**b**) phase angle of the impedance. The typical (**c**) cyclic voltammetry (CV).

Figure 5 shows the electrochemical characteristic of the stimulation electrodes. The EIS measurement revealed that the average magnitude of the impedance at 1 kHz was 1468 ± 333.3 Ω, whose value lies in the order of kiloohms, which is reasonable for the extracellular stimulation electrodes [32]. The average phase angle was −63 ± 4.3°, indicating that a fabricated spinal cord electrode delivers charge via capacitive and faradaic processes. The charge storage capacity (CSC) of the fabricated electrodes was calculated as 33.1 ± 13.4 μC/cm^2^.

### 3.3. In Vivo Evaluation

**Figure 6:**
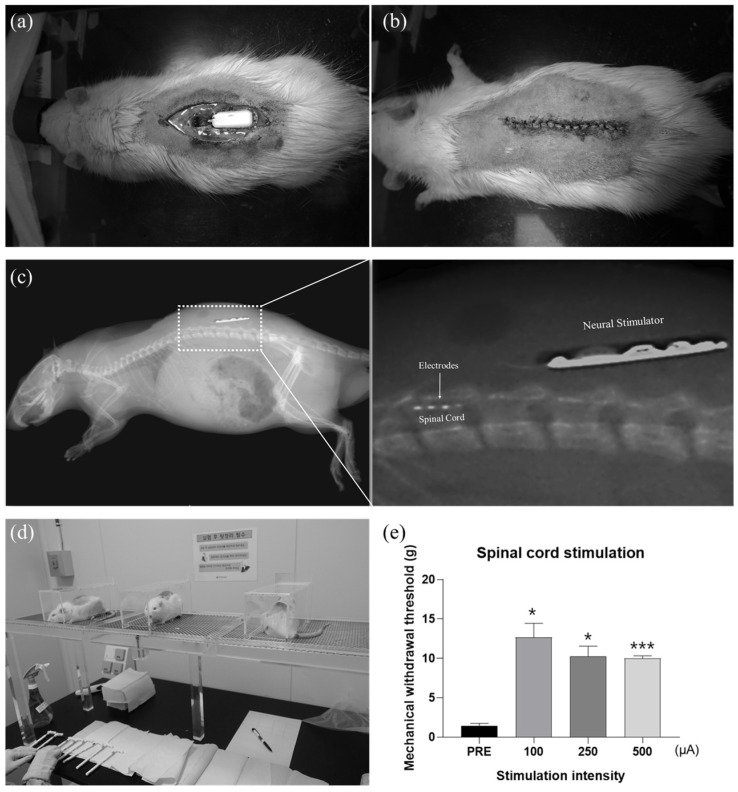
(**a**) Surgical implantation of a neural stimulator in the rat model of neuropathic pain. (**b**) Photograph of the rat after surgery, and (**c**) confirmation of implantation using X-ray images. (**d**) Experimental setup for Von Frey test to determine thresholds of the flexion withdrawal reflex in response to mechanical stimulation of the left hind limb. (**e**) Verification of the spinal cord stimulation effect. Measured mechanical withdrawal threshold according to the stimulation intensity (* *p* < 0.05, *** *p* < 0.005).

The neural stimulator was implanted above the spine (Figure 6a–c), and its electrodes are placed on the spinal cord, as shown in the X-ray image (Figure 6c), confirming its location after surgery. After placing animals into the cage for the Von Frey test, behavioral responses were observed for both control and experimental groups. In the control experiment, behavioral responses of rats were observed, while no electrical stimulation was applied. The paw withdrawal threshold was 1.47 ± 0.279 g, which shows an extremely hypersensitive state. However, after electrical stimulation with different parameters, the response threshold was dramatically increased to 12.7 ± 1.79 g, 10.3 ± 1.27 g, and 9.99 ± 0.328 g, respectively. Although the threshold tends to slightly decrease as the stimulation intensity increases, it still obviously shows pain-relieving effects in the model. Notably, at 100 μA, the threshold almost even returned to normal.

## 4. Discussion and Conclusions

In this study, a fully implantable LCP-based miniaturized neural stimulator for spinal cord stimulation was developed. The electrode, the package, and the substrate for the circuitries are microfabricated using the LCP and monolithically integrated, enabling miniaturization of the entire device (device size of 0.447 cm^3^ and the electrode thickness of 100 μm). The functionality of the remote controller, the external relay, and the neural stimulator was tested, and the characteristics of the electrodes were evaluated in vitro. Paw withdrawal response was evaluated by an in vivo von Frey test and showed a remarkably reduced pain response. The mechanical withdrawal threshold was increased after electrical stimulation, and it even brought them back to a normal state at the certain parameter, confirming the effectiveness of the spinal cord stimulation in pain control.

Our LCP-based spinal cord stimulator has several advantages over the conventional ones. The metal packaged conventional spinal cord stimulation system has a separate IPG and an electrode array, which are connected by the long lead wire. Due to their modular configuration, moisture can smear into the gap at the binding site of each module, shortening the lifetime. However, since our device is monolithically fabricated and packaged by LCP having extremely low moisture absorption properties (<0.04%), we could eliminate the moisture leakage path at the binding site of each module. When comparing LCP with other biocompatible polymers used to develop a neural probe, LCP offers lower moisture absorption than others such as polyimide (2.8%) and parylene-C (0.06–0.6%) [25]. The long-term reliability of the LCP encapsulation including LCP-based electrode arrays and the LCP packaging of electronics has been demonstrated in the previous studies. It was first investigated in [31] as an encapsulation material of the microelectrode array for retinal implants, resulting in 2 months of accelerated soak test at 75 °C with no degradation in LCP–LCP adhesion, and 3 months of in vivo test in rabbit eyes with no chorioretinal inflammation. It was demonstrated as an encapsulation of the radio frequency integrated circuit, showing stable RF characteristics in vivo for more than 40 days and an expected device lifetime of more than 2 years in in vitro accelerated soak tests [33]. It was also tested as a base material of the monolithic retinal prosthesis. At least 8 years of the device lifetime (LCP–LCP interface, LCP–metal interface, and LCP surface permeation included) was shown in in vitro accelerated soak tests, and stability in in vivo for 2.5 years [37]. Lastly, the chronic reliability of an encapsulation material of micro-electrocorticographic (µECoG) array was demonstrated, resulting in at least 3.4 years of lifetime in in vitro accelerated soak tests [38]. In addition, a fabrication method to enhance the adhesion of metal–polymer was studied, showing 21% enhanced mean time to failure in in vitro tests [35].

Since the LCP-based electrodes are manufactured based on a semiconductor microfabrication process, the electrode can be fabricated with high precision and easily customized based on the patients and applications. The LCP-based spinal cord stimulator can be readily extended and utilized for a much wider range of implantable neural stimulator such as deep-brain stimulation, brain–machine interface, and other sensory and motor prosthetics, opening a new avenue toward miniaturized polymer-based neural prosthetics.

## Data Availability

Not applicable.
